# Angiotensin II affects inflammation mechanisms via AMPK-related signalling pathways in HL-1 atrial myocytes

**DOI:** 10.1038/s41598-017-09675-3

**Published:** 2017-09-04

**Authors:** Nami Kim, Youngae Jung, Miso Nam, Mi Sun Kang, Min Kyung Lee, Youngjin Cho, Eue-Keun Choi, Geum-Sook Hwang, Hyeon Soo Kim

**Affiliations:** 1 Integrated Metabolomics Research Group, Western Seoul Center, Korea Basic Science Institute, Seoul, 120-140 Republic of Korea; 2 Division of Cardiology, Department of Internal Medicine, Seoul National University Bundang Hospital, Seongnam, Republic of Korea; 3 Division of Cardiology, Department of Internal Medicine, Seoul National University Hospital, Seoul, Republic of Korea; 4 Chemistry & Nanoscience, Ewha Womans University, Seoul, Republic of Korea; 5 Department of Anatomy, Korea University College of Medicine, Seoul, 02841 Republic of Korea

## Abstract

Inflammation is a common cause of cardiac arrhythmia. Angiotensin ІІ (Ang ІІ) is a major contributing factor in the pathogenesis of cardiac inflammation; however, its underlying molecular mechanism remains unclear. Here, we explored the effect of Ang ІІ on inflammatory mechanisms and oxidative stress using HL-1 atrial myocytes. We showed that Ang ІІ activated c-Jun N-terminal kinase (JNK) phosphorylation and other inflammatory markers, such as transforming growth factor-β1 (TGF-β1) and tumor necrosis factor-α (TNF-α). Ang ІІ decreased oxygen consumption rate, which resulted in reactive oxygen species (ROS) generation and inhibition of ROS blocked Ang II-mediated JNK phosphorylation and TGF-β1 induction. Ang ІІ induced the expression of its specific receptor, AT1R. Ang II-induced intracellular calcium production associated with Ang ІІ-mediated signalling pathways. In addition, the generated ROS and calcium stimulated AMPK phosphorylation. Inhibiting AMPK blocked Ang II-mediated JNK and TGF-β signalling pathways. Ang ІІ concentration, along with TGF-β1 and tumor necrosis factor-α levels, was slightly increased in plasma of patients with atrial fibrillation. Taken together, these results suggest that Ang ІІ induces inflammation mechanisms through an AMPK-related signalling pathway. Our results provide new molecular targets for the development of therapeutics for inflammation-related conditions, such as atrial fibrillation.

## Introduction

Atrial fibrillation is a clinically common sustained type of cardiac arrhythmia with high morbidity and mortality that occurs through structural and electrical remodelling due to conditions such as heart failure and fibrosis^1, 2^. General treatment options such as medicines, medical procedures, and lifestyle changes are available. For rhythm control, drugs, including aspirin, warfarin, amiodarone, and beta blockers, are typically the first choice^3–5^. Additionally, medical procedures such as electrical cardioversion, catheter ablation, and open-heart surgery are second-line choices^6, 7^. Nevertheless, the relapse rate after drug treatment or operation is high because the precise physiological mechanism of atrial fibrillation remains unclear.

In the renin-angiotensin system, angiotensin ІІ (Ang ІІ) regulates cardiac remodelling during atrial fibrillation^8^. In addition, Ang ІІ is implicated in different cardiovascular diseases such as hypertension, atherosclerosis, and heart failure^9^. Furthermore, Ang II controls cardiac contractility, cell coupling, and impulse propagation through activation of the Ang ІІ type 1 receptor (AT1R), a specific Ang II receptor^10^.

Ang II induces reactive oxygen species (ROS) generation, which activates multiple intracellular second messenger molecules such as mitogen-activated protein kinases (MAPK), transforming growth factor-β1 (TGF-β1), nuclear factor-κB (NF-κB), and cytokines, including interleukin-1β (IL-1β), interleukin-6 (IL-6), and tumor necrosis factor α (TNFα)^11, 12^. The MAPK pathway, including c-Jun N-terminal kinase (JNK) activation, is associated with various pathological conditions such as cancer, stroke, inflammatory disease, and heart failure^13, 14^. Furthermore, TGF-β1 contributes to cardiac remodelling through oxidative stress by activation of NAD(P)H oxidase^12^. NF-κB, a transcription factor that is a critical molecule in atrial fibrillation pathogenesis, is associated with inflammation, an important contributor to atrial fibrillation^15^.

Recent studies indicated that arrhythmia is associated with the calcium-dependent pathway by activating calcium/calmodulin-dependent protein kinase kinase (CaMKK) and directly regulates the AMP-activated protein kinase (AMPK) pathway^16, 17^. AMPK is an energy sensor that activates energy producing metabolic pathways and inhibits energy consuming pathways, including biosynthesis, cell growth, and cell proliferation^16, 18^. AMPK is activated in response to the increased AMP/ATP ratio that occurs under metabolic stresses such as hypoxia, starvation, glucose deprivation, and muscle contraction^19^. AMPK is especially important in the heart, which requires a large amount of energy compared to other organs. Consequently, AMPK contributes to the critical regulation of cardiac energy status^20^. AMPK activation by alleviating metabolic cellular stress plays a critical role in cellular myocardial dysfunction^16, 20^. Although the specific molecular mechanism underlying AMPK regulation during atrial fibrillation remains unclear, it is believed to regulate abnormal cardiac contraction, fibrosis, and arrhythmia.

In the present study, we investigated the mechanism underlying atrial inflammation using HL-1 atrial cells after treatment with Ang ІІ, which is known to cause structural and electrical remodeling^21^. Interestingly, our results showed that Ang ІІ induced inflammation, resulting in the activation of JNK, TGF-β1, NF-κB, and AMPK by generating ROS through AT1R. To further confirm this hypothesis in human atrial fibrillation, we detected Ang II and inflammatory cytokines in the plasma of patients with atrial fibrillation. Interestingly, Ang ІІ concentrations and inflammatory cytokine levels were slightly increased in the plasma of patients with atrial fibrillation. Together, these results suggest that Ang II may regulate atrial fibrillation through activating inflammatory mechanisms and the AMPK signalling pathway via ROS generation.

## Results

### Ang ІІ induces inflammation mechanisms in HL-1 atrial cells

**Figure 1:**
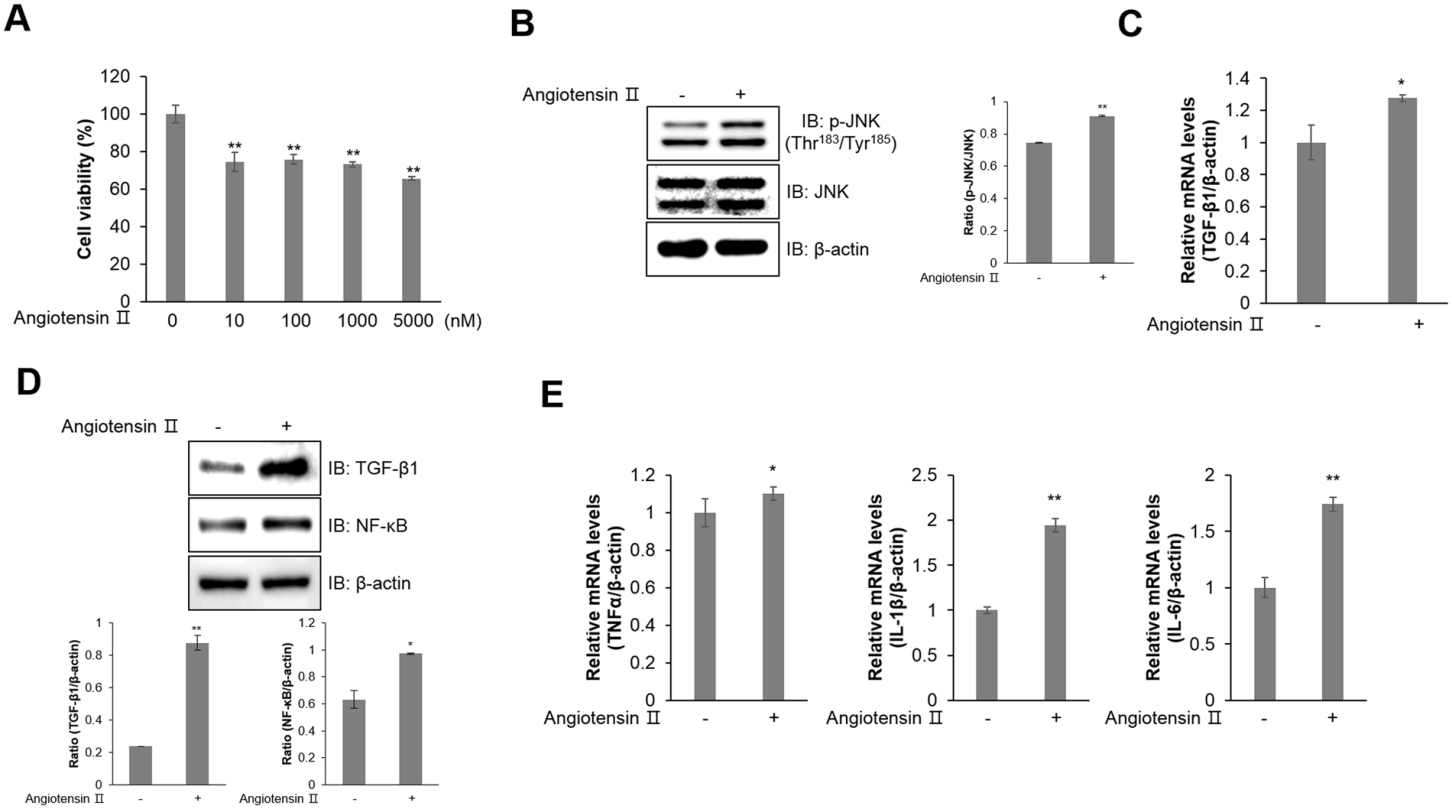
Ang ІІ induces inflammation mechanism in HL-1 atrial cells. (**A**) HL-1 cells were treated with the indicated doses of Ang ІІ for 24 h. Cell viability was analysed with an MTT assay. **(B)** Cells were stimulated with 1 μM Ang ІІ for 24 h. Cells were then lysed, and JNK phosphorylation was quantified by western blot analysis using an antibody specific to the phosphorylated protein. The levels of total JNK and β-actin were also measured as protein loading controls. **(C)** Total RNA was isolated from Ang ІІ-treated HL-1 cells, and quantitative RT-PCR analysis was performed with specific primers targeted to *Tgf-β1* and *β-actin* genes. *β-actin* was used as an endogenous control. **(D)** The protein expression of TGF-β1, NF-κB, and β-actin was evaluated by western blot analysis. Blotting with an antibody specific to β-actin served as a control. **(E)** Relative mRNA levels of *Tnfα, Il-1β*, and *Il-6* were measured by quantitative RT-PCR with target genes. *β-actin* was used as an endogenous control. *P < 0.05, **P < 0.01, compared with the untreated controls. Results are from three independent experiments.

To understand the inflammatory mechanism of Ang ІІ in atrial cells, we treated atrial HL-1 cells with Ang ІІ, First, we performed the MTT assay with Ang ІІ. The cell viability of HL-1 cells was slightly decreased upon treatment of up to 5 μM of Ang ІІ (Fig. 1A). Administration of Ang ІІ induced JNK phosphorylation in HL-1 cells (Fig. 1B). Results from real-time RT-PCR and western blot analysis showed that Ang ІІ treatment increased mRNA and protein levels of TGF-β1 (Fig. 1C and D). Another inflammatory marker, NF-κB protein level was markedly increased by Ang ІІ treatment (Fig. 1D). Other inflammatory markers, such as TNFα, IL-1β, and IL-6. Their relative mRNA levels were increased in Ang ІІ-treated HL-1 cells (Fig. 1E). These results suggest that Ang ІІ induces the activation of inflammation via JNK, TGF-β1, NF-κB, and their downstream mediators.

### Ang ІІ regulates mitochondrial oxygen consumption and generates ROS in HL-1 atrial cells

**Figure 2:**
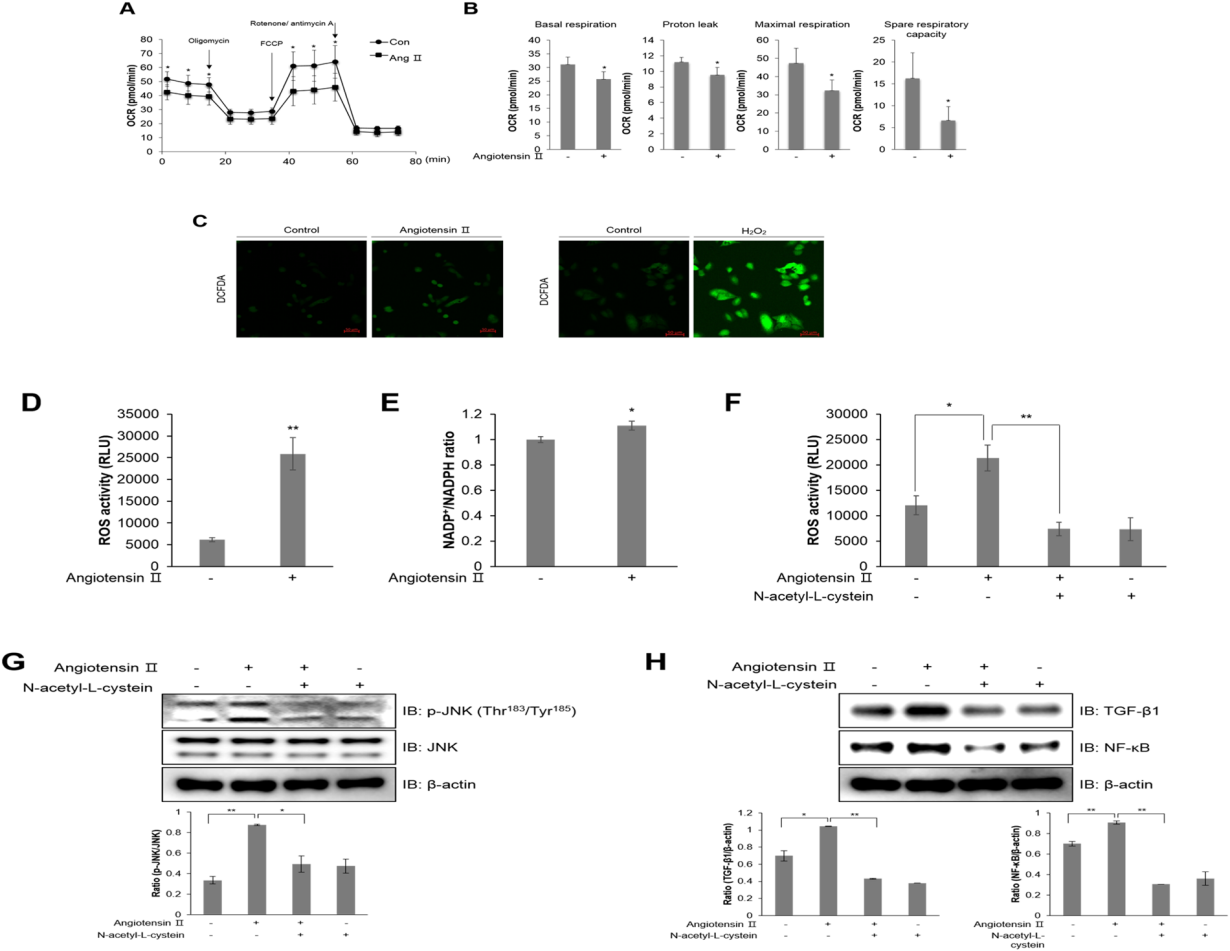
Ang ІІ regulates mitochondrial oxygen consumption and generates ROS in HL-1 atrial cells. (**A**) Mitochondrial oxygen consumption rate (OCR) in HL-1 cells was measured using the Seahorse flux analyzer in response to 1 μM oligomycin, 1 μM FCCP, and 0.5 μM rotenone/antimycin (**A)**. (**B**) Basal respiration, proton leak, maximal respiration, and spare respiratory capacity were calculated from (**A**). (**C**) HL-1 cells were pre-treated with 10 μM DCFDA for 40 min, and then treated with 1 μM Ang ІІ and 100 μM H_2_O_2_, respectively. H_2_O_2_ was used as a positive control. ROS levels were assessed using a confocal microscope in real-time. (**D**) HL-1 cells were treated with 1 μM Ang ІІ for 24 h. The cells were lysed with ROS detection solution, and the concentration of ROS was evaluated by colorimetric absorbance assay. (**E**) HL-1 cells were stimulated with 1 μM Ang ІІ for 24 h, and the NADP^+^/NADPH ratio was calculated by NADP^+^/NADPH colorimetric assay. HL-1 cells were pre-treated with 10 mM N-acetyl-L-cysteine (NAC) for 30 min and then treated with 1 μM Ang ІІ for 24 h. (**F**) ROS levels as measured by a colorimetric absorbance assay. (**G**) JNK phosphorylation was quantified by western blot analysis using antibodies specific to the phosphorylated protein. Levels of total JNK and β-actin were also measured as protein loading controls. **(H)** TGF-β1 and NF-κB protein levels were assessed by immunoblotting with specific antibodies. *P < 0.05, **P < 0.01, compared with the untreated cells. Results are from three independent experiments.

A functional mitochondrial angiotensin system was previously identified and characterized^22^. The precise mechanisms mediating the modulation of mitochondrial respiration by Ang II are not known. To identify the mechanism underlying atrial inflammation, we examined how Ang ІІ affects mitochondrial respiration in HL-1 cells. Representative data for the extracellular mitochondrial flux analysis showed that Ang ІІ treatment for 24 h decreased the basal oxygen consumption rate (OCR) in these cells (Fig. 2A). Furthermore, administering FCCP, a mitochondrial membrane uncoupler, significantly decreased OCR levels compared to control levels. The XF data measured 4 different mitochondrial parameters, including basal respiration, proton leak, maximal respiration, and spare respiratory capacity (Fig. 2B). All these parameters were decreased upon Ang ІІ treatment. This downregulation of mitochondria electron transport activity is correlated with mitochondria dysfunction by oxidative stress during atrial inflammation^23^. To understand the involvement of ROS in atrial inflammation, we quantified ROS levels in HL-1 cells upon Ang ІІ treatment using DCFDA dye (Fig. 2C). Ang ІІ treatment increased the green fluorescence intensity resulting from DCFDA oxidation in HL-1 cells. To examine the effect of Ang ІІ on mitochondria and cellular ROS production, we also measured ROS activity in Ang ІІ-stimulated cells (Fig. 2D). NADP^+^/NADPH levels were assessed to evaluate NADPH oxidase activity during ROS generation (Fig. 2E). Ang ІІ increased the NADP^+^/NADPH ratio in HL-1 cells. However, Ang ІІ-stimulated ROS generation was blocked by N-acetyl-L-cysteine (NAC), a ROS scavenger, in HL-1 cells (Fig. 2F). Furthermore, Ang ІІ-induced JNK phosphorylation was downregulated by NAC treatment in HL-1 cells (Fig. 2G). The induction of NF-κB and TGF-β1 expression was also blocked by NAC treatment in Ang ІІ-treated HL-1 cells (Fig. 2H). Altogether, these results indicated that Ang ІІ regulates mitochondria activity through intracellular ROS generation and inflammation mechanisms are subsequently induced in atrial cells.

### Ang ІІ induces AT1R expression in HL-1 atrial cells

**Figure 3:**
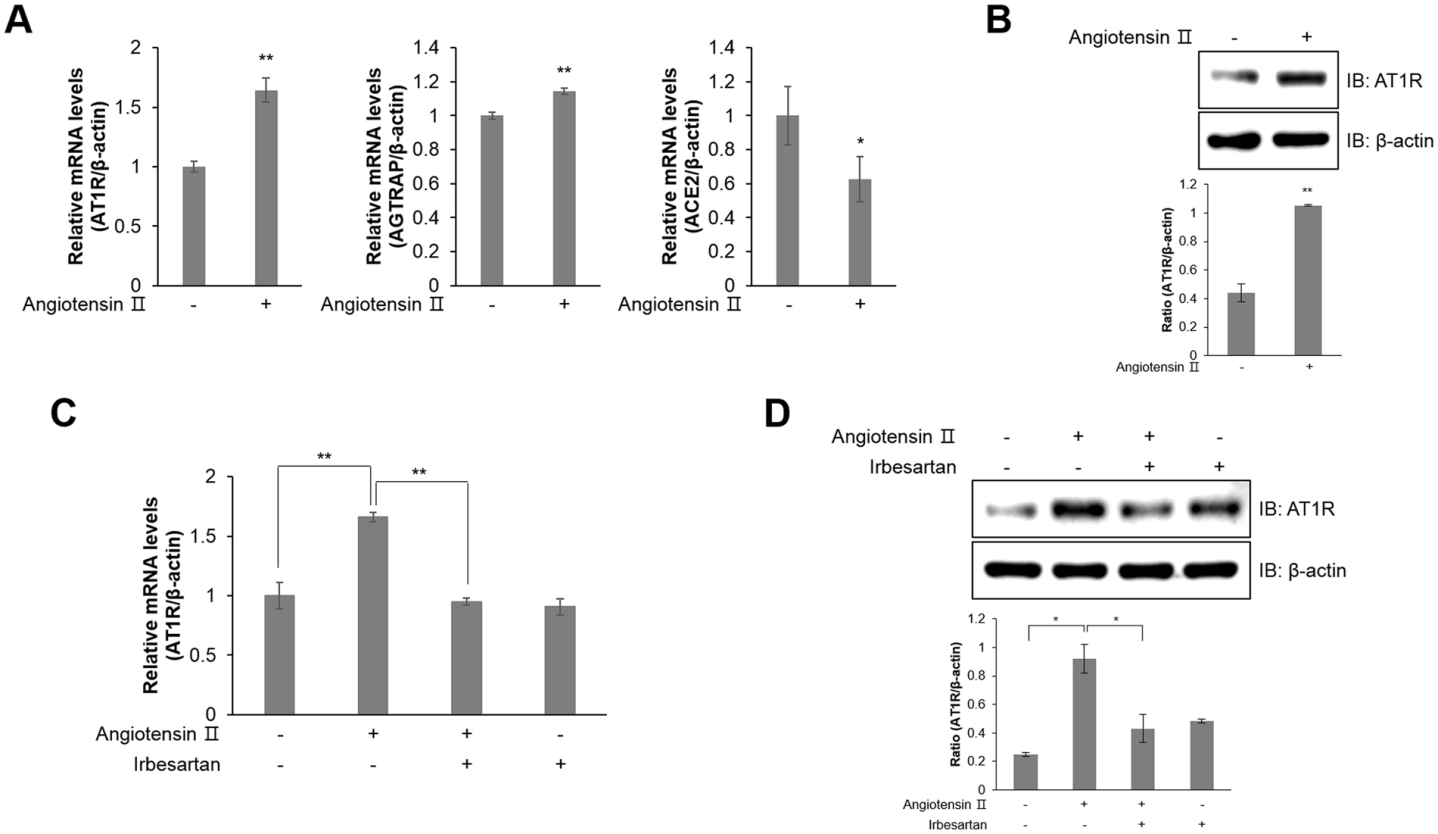
Ang ІІ induces AT1R expression in HL-1 atrial cells. (**A**) HL-1 cells were treated with Ang ІІ (1 μM) for 24 h. Total mRNA was isolated from each sample, and quantitative RT-PCR analysis was performed with specific primers targeted to *At1r, Agtrap, Ace2*, and *β-actin*; *β-actin* was used as an endogenous control. (**B**) Ang ІІ-stimulated cells were lysed and AT1R protein expression was quantified by western blot analysis. Blotting with antibodies specific to β-actin served as a loading control. (**C**) HL-1 cells were pre-treated with irbesartan (1 μM) for 30 min and treated with Ang ІІ for 24 h. Quantitative RT-PCR was performed with specific primers targeted to *At1r* and *β-actin*; *β-actin* was used as an endogenous control. (**D**) AT1R protein levels were assessed by western blot analysis with a specific antibody. Blotting with an antibody specific to β-actin served as a loading control. *P < 0.05, **P < 0.01, compared with the untreated controls. Results are from three independent experiments. Blots are displayed in cropped format.

Among G protein-coupled receptors (GPCRs), AT1R contributes to vascular remodelling, inducing hypertrophy, hyperplasia, and ROS generation^24^. Ang ІІ treatment increased the relative mRNA expression of AT1R and Ang II receptor-associated protein (AGTRAP), while decreasing the levels of angiotensin-converting enzyme 2 (ACE2), which disrupts ROS production (Fig. 3A). Furthermore, Ang ІІ treatment activated AT1R protein expression (Fig. 3B). In contrast, AT1R relative mRNA (Fig. 3C) and protein (Fig. 3D) expression was downregulated in HL-1 cells treated with 1 μM irbesartan, an angiotensin receptor blocker. These results suggest that Ang ІІ induces AT1R expression in atrial cells.

### Blocking the angiotensin receptor prevents inflammatory responses by suppressing ROS generation in HL-1 atrial cells

**Figure 4:**
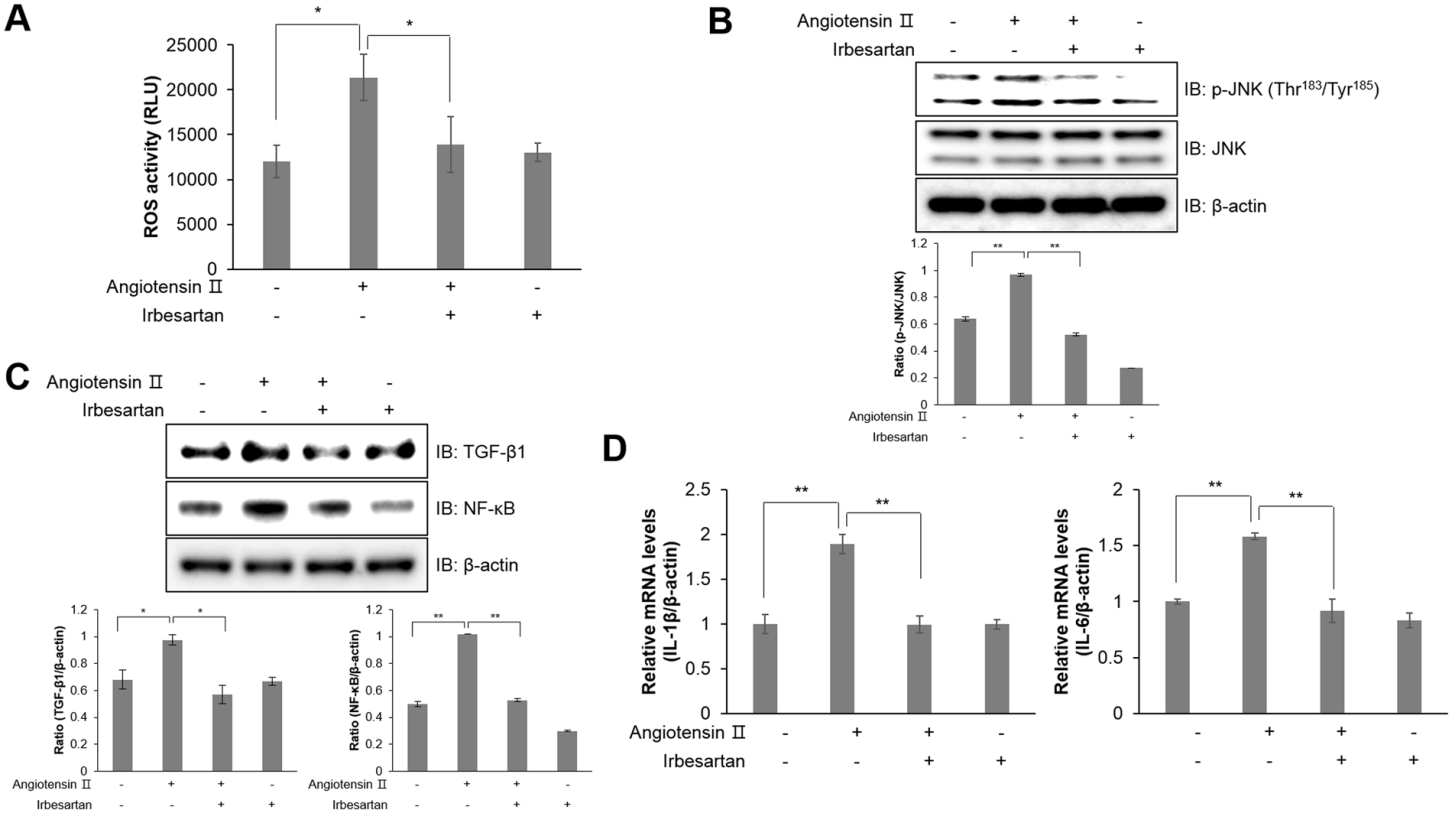
Angiotensin receptor blockade prevents inflammatory responses by suppressing ROS generation in HL-1 atrial cells. (**A**) HL-1 cells were treated with 1 μM irbesartan for 30 min and stimulated with Ang ІІ for 24 h. The intracellular ROS concentration was detected by a colorimetric absorbance assay. (**B**) JNK phosphorylation and β-actin were quantified by western blot analysis. The level of β-actin was measured as a protein loading control. (**C**) Protein expression of TGF-β1, NF-κB, and β-actin was evaluated by western blot analysis. Blotting with an antibody specific to β-actin served as a control. (**D**) Total mRNA was isolated from each sample, and quantitative RT-PCR was performed with specific primers targeted to *Il-1β*, *Il-6*, and *β-actin*; *β-actin* was used as an endogenous control. *P < 0.05, **P < 0.01, compared with the untreated controls. Results are from three independent experiments. Blots are displayed in cropped format.

To determine whether Ang ІІ induced atrial inflammation through AT1R, we performed a ROS activity assay in the presence of irbesartan. Following treatment with Ang ІІ, ROS generation was suppressed by irbesartan (Fig. 4A). AT1R blockade by irbesartan treatment inhibited ROS generation in Ang ІІ-treated HL-1 cells. In addition, irbesartan treatment also abolished JNK phosphorylation upon Ang ІІ exposure (Fig. 4B). Moreover, pretreatment with irbesartan downregulated TGF-β1 and NF-κB expression in Ang ІІ-treated HL-1 cells (Fig. 4C). Ang ІІ treatment increased relative mRNA levels of *Il-1β* and *Il-6*, which are risk factors for atrial inflammation, but this effect was blocked by irbesartan treatment (Fig. 4D). Thus, these results indicated that Ang ІІ induced inflammation by ROS generation via AT1R.

### Cellular calcium is involved in Ang ІІ-induced inflammation

**Figure 5:**
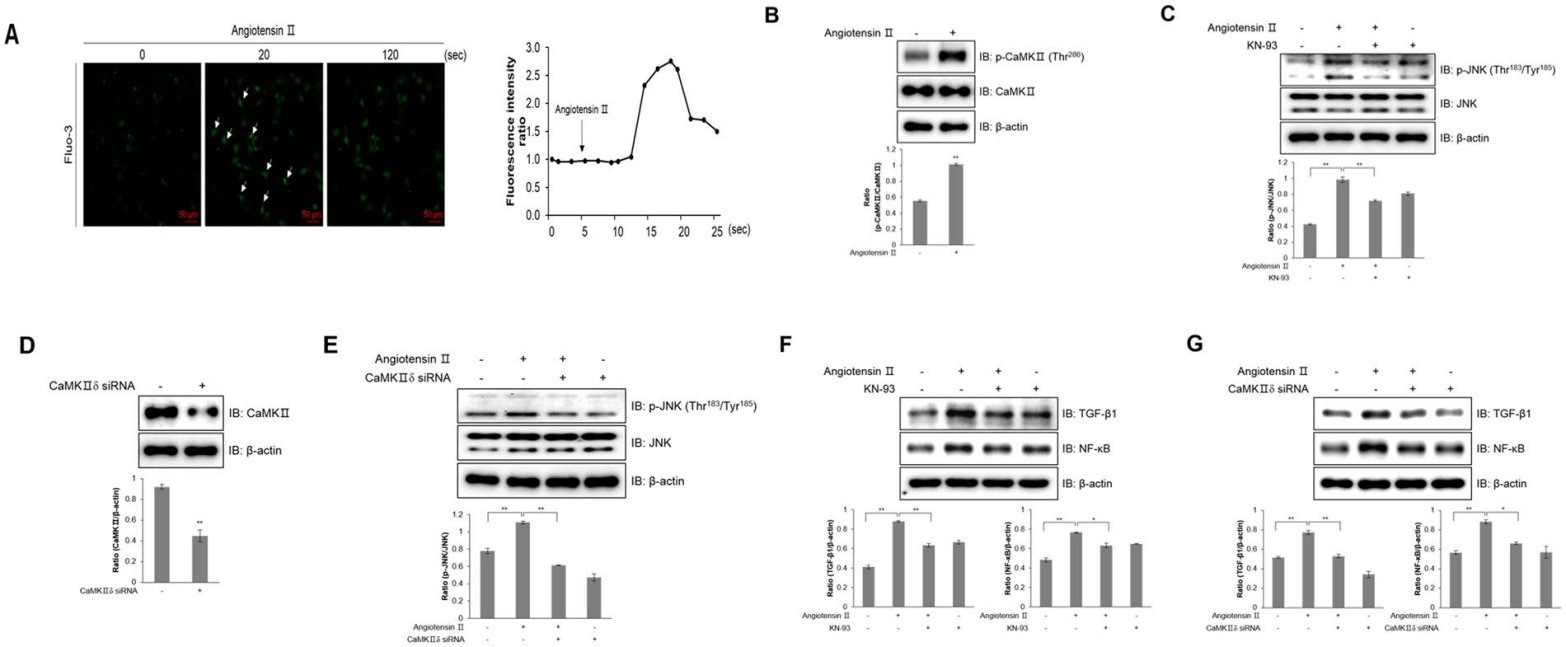
Cellular calcium is involved in Ang ІІ-induced inflammation. (**A**) HL-1 cells were pre-treated with fluo-3 AM for 30 min and then with 1 μM Ang ІІ. Green fluorescence was detected using a confocal microscope. (**B**) HL-1 cells were treated with Ang ІІ (1 μM) for 24 h. Phosphorylated CaMKІІ and CaMKІІ protein levels were assessed by western blot analysis with a specific antibody. (**C**) Cells were pre-treated with KN-93 (5 μM) for 30 min and treated with Ang ІІ for 24 h. Cell lysates were analysed by western blotting analysis with antibodies against phosphorylated JNK. Non-phosphorylated JNK was used as a control. β-actin served as a protein loading control. (**D**) Cells were transiently transfected with CaMKІІδ siRNA (100 nM) for 2 days and then treated with Ang ІІ (1 μM) for 24 h. CaMKІІδ protein levels were assessed by western blot analysis with a specific antibody. (**E**) HL-1 cells were transiently transfected with CaMKІІδ siRNA and then the cells were lysed, and the expression of p-JNK, JNK, and β-actin was quantified by western blot analysis. Levels of JNK and β-actin were also measured as protein loading controls. (**F**) Cells were treated with KN-93 and then incubated with Ang ІІ. The protein expression of TGF-β1, NF-κB, and β-actin was evaluated by western blot analysis. Blotting with an antibody specific to β-actin served as a control. (**G**) CaMKІІδ siRNA was transiently transfected into the cells, followed by Ang ІІ treatment. The cell lysates were analysed by western blot analysis with TGF-β1, NF-κB, and β-actin antibodies. *P < 0.05, **P < 0.01, compared with the untreated controls. Results are from three independent experiments. Blots are displayed in cropped format.

AT1R stimulation increases intracellular calcium^25^. To investigate whether intracellular calcium was involved in atrial inflammation, we measured the intracellular calcium concentration using fluo-3 AM, a calcium indicator. Ang ІІ increased the green fluorescence intensity representative of calcium concentration in HL-1 cells (Fig. 5A). This increase of calcium concentration was not significantly affected in the presence of thapsigargin, an ER calcium depleting agent. Ang II-induced calcium increases were dramatically suppressed by conducting experiments in calcium-free medium, indicating that increased intracellular calcium levels resulted mainly from extracellular calcium influx, rather than intracellular calcium stores. Next, we examined the involvement of calcium/calmodulin-dependent protein kinase ІІ (CaMKІІ). Ang ІІ significantly increased CaMKІІ phosphorylation in HL-1 cells (Fig. 5B). To confirm this result, we pretreated HL-1 cells with KN-93, a CaMKІІ inhibitor. KN-93 attenuated Ang ІІ-induced JNK phosphorylation (Fig. 5C). To further assess the effect of CaMKІІ on inflammation, we knockdowned CaMKІІδ by transfecting with 100 nM siRNA (Fig. 5D). Knockdown of CaMKІІδ also downregulated JNK phosphorylation upon Ang ІІ treatment (Fig. 5E). Additionally, KN-93 treatment and knockdown of CaMKІІδ blocked the protein expression of TGF-β1 and NF-κB (Fig. 5F and G), suggesting that Ang ІІ induces inflammation through a calcium-mediated signalling pathway.

### Ang ІІ activates inflammation through AMPK in HL-1 atrial cells

**Figure 6:**
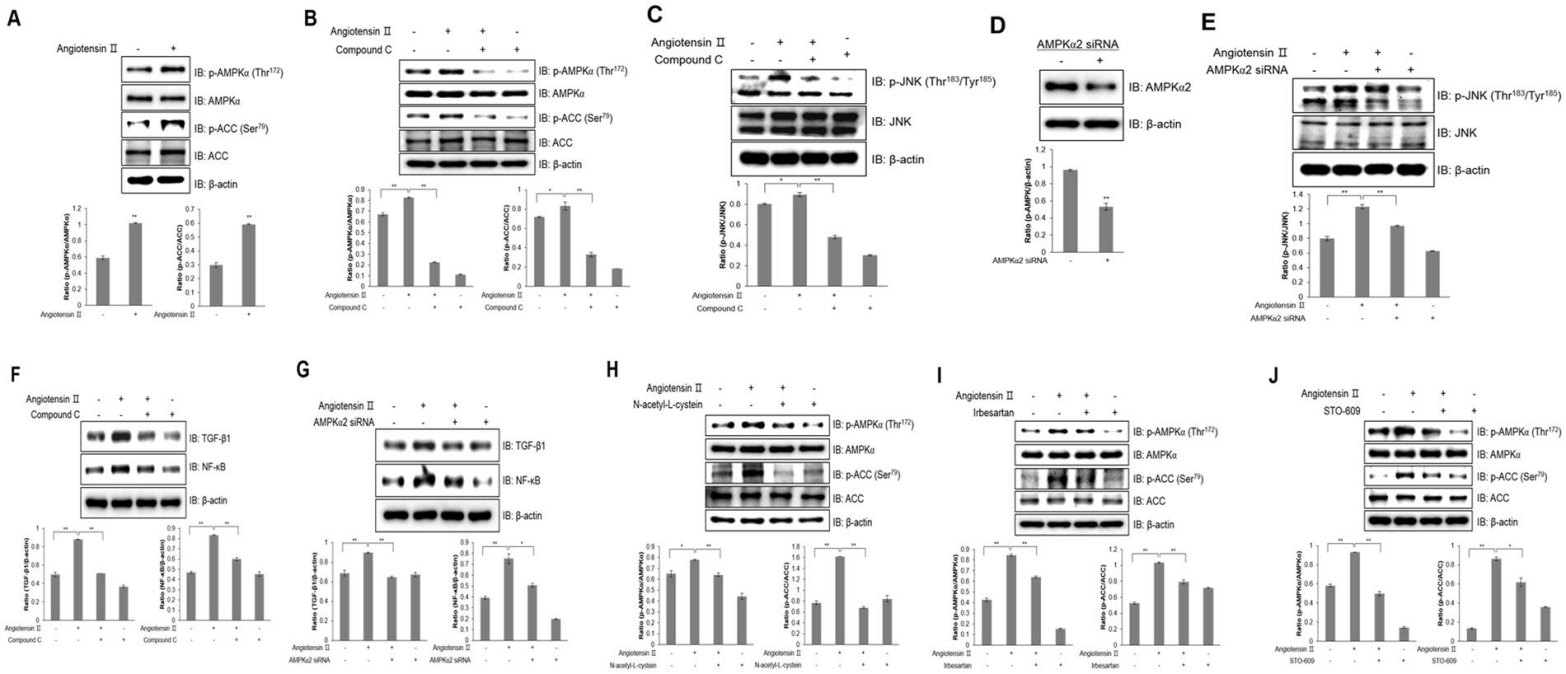
Ang ІІ activates inflammation through AMPK in HL-1 atrial cells. (**A**) HL-1 cells were stimulated with 1 μM Ang ІІ for 24 h. Cell lysates were analysed by western blotting with antibodies against phosphorylated AMPKα2 and ACC. Levels of total AMPK and ACC were used as controls, and β-actin served as a protein loading control. (**B**) HL-1 cells were pre-treated with compound C (5 μM) for 30 min and treated with Ang ІІ for 24 h. AMPKα and ACC phosphorylation was quantified by western blot analysis using antibodies specific to the phosphorylated proteins. Total AMPK, ACC, and β-actin levels were measured as controls for protein loading. (**C**) Cells were stimulated with Ang ІІ for 24 h. Proteins were immunoprecipitated with the AMPKα antibody. Protein expression was analysed via western blot analysis by using JNK antibody. (**D**) HL-1 cells were stimulated with Ang ІІ in the presence of compound C. The protein expression of phosphorylated JNK, total JNK, and β-actin was analysed by western blotting. Blotting with antibodies specific to JNK and β-actin served as controls. (**E**) HL-1 cells were transiently transfected with AMPKα2 siRNA (100 nM) for 2 days and then treated with Ang ІІ (1 μM) for 24 h. Cell lysates were assessed by western blotting with AMPK and β-actin antibodies. (**F**) AMPKα2 siRNA was transiently transfected into cells followed by Ang ІІ treatment. Cells were lysed, and p-JNK, JNK, and β-actin expression levels were quantified by western blotting. JNK and β-actin levels were also measured as protein loading controls. (**G**) HL-1 cells were pre-treated with compound C for 30 min and treated with Ang ІІ for 24 h. TGF-β1 and NF-κB expression levels were quantified by western blot analysis using antibodies specific to the phosphorylated proteins. β-actin level was measured as a protein loading control. (**H**) HL-1 cells were transiently transfected with AMPKα2 siRNA and treated with Ang ІІ. Cell lysates and TGF-β1, NF-κB, and β-actin levels were analysed by western blotting. (**I**) HL-1 cells were treated with Ang ІІ in the presence of NAC. Protein expression of phosphorylated AMPKα and ACC was measured by immunoblotting with specific antibodies to the phosphorylated proteins. Total AMPK, ACC, and β-actin levels were quantified as controls. (**J**) HL-1 cells were stimulated with Ang ІІ in the presence of irbesartan. AMPKα and ACC phosphorylation was assessed by western blotting using specific antibodies, and total AMPKα, ACC, and β-actin levels were quantified as controls. (**K**) HL-1 cells were pre-treated with STO-609 and then treated with Ang ІІ. Cell lysates were analysed by western blotting with antibodies against phosphorylated AMPKα and ACC. Non-phosphorylated AMPKα and ACC were used as controls. β-actin served as a protein loading control. *P < 0.05, **P < 0.01, compared with the untreated cells. Results are from three independent experiments. Blots are displayed in cropped format.

Atrial inflammation is associated with metabolic stress, which activates AMPK downstream of CaMKK, which is regulated by calcium^26, 27^. Therefore, we hypothesized that Ang ІІ controls atrial inflammation through activating the AMPK signalling pathway. To confirm this hypothesis, we examined AMPK activation. Ang ІІ increased AMPKα phosphorylation in HL-1 cells as well as phosphorylation of ACC, a downstream target of AMPK (Fig. 6A). Inhibiting AMPK by compound C treatment abolished Ang II-induced phosphorylation of ACC (Fig. 6B). To characterize the correlation between AMPK and JNK activation, we then examined whether AMPK directly interact with JNK in Ang ІІ-treated HL-1 cells.

The signal of co-immunoprecipitated JNK increased upon Ang II treatment, indicating that Ang II induces direct interaction between AMPK with JNK (Fig. 6C). Consistently, we examined the level of JNK phosphorylation in HL-1 pretreated with compound C and showed that JNK phosphorylation was abrogated by AMPK inhibition (Fig. 6D). We also used AMPKα2 siRNA to knockdown. Transfecting HL-1 cells with 100 nM AMPKα2 siRNA markedly downregulated protein expression of AMPKα (Fig. 6E). AMPKα2 knockdown blocked Ang ІІ-induced JNK phosphorylation (Fig. 6F). After pre-treatment with compound C (Fig. 6G) and AMPKα2 knockdown (Fig. 6H), Ang ІІ treatment no longer had any effect on TGF-β1 and NF-κB. Moreover, phosphorylation of AMPKα and its downstream partner, ACC, was blocked in Ang ІІ-stimulated HL-1 cells pre-treated with NAC, a ROS scavenger (Fig. 6I). To assess the effect of AT1R, we blocked AT1R activation by irbesartan treatment. AT1R blockade suppressed AMPKα signalling pathway activation (Fig. 6J). Furthermore, STO-609, CaMKK inhibitor, treatment abolished AMPK and ACC phosphorylation in HL-1 cells (Fig. 6K). Together, these results suggest that Ang II induces inflammation through activation of the AT1R-calcium-AMPK-JNK axis pathway in atrial cells.

### Plasma Ang ІІ is increased in patients with atrial fibrillation

**Figure 7:**
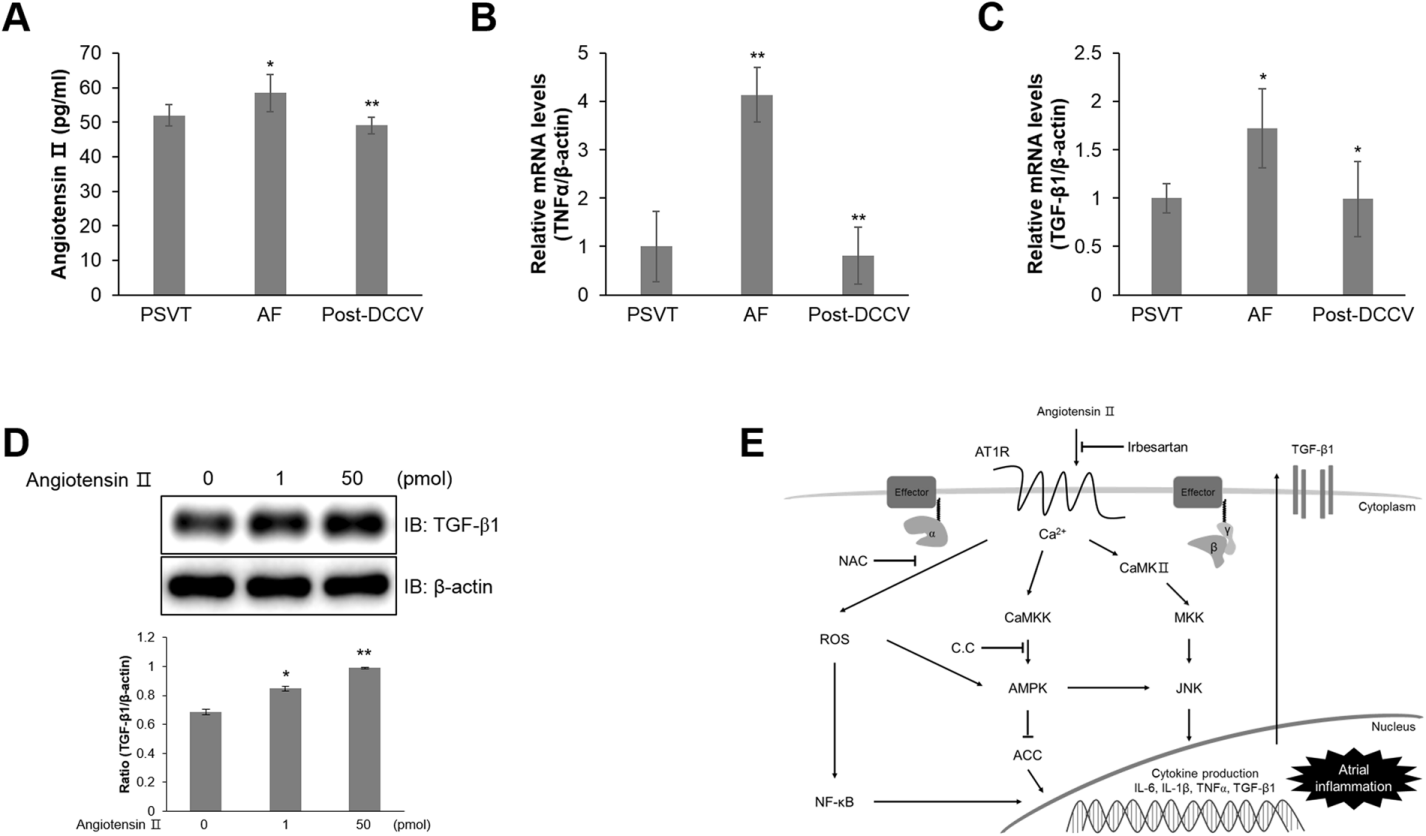
Ang ІІ is increased in patients with atrial fibrillation. (**A**) The concentration of Ang ІІ in plasma samples from patients with PSVT (n = 6), DCCV (n = 11), and post-DCCV (n = 11). (**B**) Total mRNA was extracted from 50 μL of plasma samples from patients with PSVT (n = 6), DCCV (n = 11), and post-DCCV (n = 11), and quantitative RT-PCR analysis was performed with specific human primers targeted to *TNFα* and *β-actin*. *β-actin* was used as an endogenous control. (**C**) Total mRNA was extracted from 50 μL of plasma samples from patients with PSVT (n = 6), DCCV (n = 11), and post-DCCV (n = 11), and quantitative RT-PCR analysis was performed with specific human primers targeted to *TGF-β1* and *β-actin*. *β-actin* was used as an endogenous control. (**D**) HL-1 cells were stimulated the indicated doses of Ang ІІ for 24 h. Cell lysates were analysed by western blot analysis using with antibody against TGF-β1. Levels of β-actin was used as control. (**E**) Schematic representation of the signalling pathway underlying atrial fibrillation. *P < 0.05, **P < 0.01 compared with the untreated cells. Results are from three independent experiments.

To get insight into the role of Ang II, we investigated the correlation between inflammation and Ang ІІ in the plasma of patients with atrial fibrillation. Patients with paroxysmal supraventricular tachycardia (PSVT) were used as controls, and patients with atrial fibrillation post-direct current cardioversion (DCCV) were enrolled. First, we measured levels of Ang ІІ in the plasma by an enzyme-linked immunosorbent assay to determine whether our *in vitro* results would be applicable to patients (Fig. 7A). Ang II concentration was slightly increased in the plasma of patients with atrial fibrillation. However, post-DCCV, plasma levels of Ang II in patients returned to a level similar to the level observed in control patients with PSVT. To confirm the inflammatory effect, we performed quantitative RT-PCR to measure relative mRNA levels of TNFα and TGF-β1 in plasma of patients (Fig. 7B and C). Furthermore, we confirmed that low concentration of Ang II affects atrial inflammation in atrial cells. The picomolar ranges of Ang II slightly increased the level of TGF-β1 (Fig. 7D). These results indicate that atrial fibrillation also correlated with inflammation induced via the Ang II. Based on our present results, we proposed a new working model for atrial inflammation (Fig. 7E).

## Discussion

In the present study, we investigated a new mechanism of angiotensin II using an atrial cell line and plasma of patients with atrial fibrillation. Ang ІІ activated inflammatory factors such as JNK, TGF-β1, and NF-κB, which induce the expression of cytokines, including TNFα, IL-1β, and IL-6 through ROS generation. Ang ІІ is known as a major bioactive peptide of the renin-angiotensin system, which controls cardiovascular homeostasis^24^. Activation of the renin-angiotensin system by Ang II increases the expression of pro-inflammatory cytokines, inflammation, and promotes the development of autoimmune dysfunction^28^. However, the precise mechanism and role of Ang ІІ on atrial fibrillation have not yet been identified. We observed the activation of inflammatory markers such as JNK, TGF-β1, and NF-κB in Ang ІІ-stimulated HL-1 atrial cells. In addition, we confirmed that the concentration of Ang ІІ was slightly increased in the plasma of patients with atrial fibrillation compared to the concentration in patients with PSVT (controls) and post-DCCV. The biological significance of this tiny increase should be studied in the future not only in basic research and but also in the clinical aspects. Additionally, the mRNA expression of TNFα and TGF-β1, representative inflammatory markers, was highly increased in patients with atrial fibrillation, and after DCCV treatment, their expression returned to normal levels. Activation of the renin-angiotensin system by Ang ІІ, which is involved in key events of the inflammatory process, plays an important role in immunologically-induced inflammation^29^. Recent studies indicated that the activation of inflammatory markers, including JNK, TGF-β1, and NF-κB, is associated with apoptosis and oxidative stress, which generates ROS, inducing inflammatory signalling pathways^30, 31^. For these reasons, we hypothesized that oxidative stress-induced activation of JNK/TGF-β1/NF-κB pathway contributes to atrial inflammation. Accordingly, we measured mitochondrial OCR in conditions of mitochondrial stress using three compounds, oligomycin, FCCP, and rotenone/antimycin A in HL-1 atrial cells. Mitochondrial parameters were significantly different between Ang ІІ-induced atrial cells and control cells. The downregulation of these parameters in Ang ІІ-induced atrial cells indicates that Ang ІІ generates ROS, resulting in the inhibition of the mitochondria electron transport chain and mitochondrial dysfunction^32, 33^. Furthermore, we investigated the importance of ROS activation during the atrial inflammation process using the atrial cells. As NADPH is an important component in the cellular anti-oxidation system, increasing of intracellular NADP^+^/NADPH ratio induces oxidative stress^34, 35^. In the Ang ІІ-treated atrial cells, NADP^+^/NADPH ratio increased and ROS generation was observed. We used NAC, a ROS scavenger, in the Ang ІІ-induced atrial cells. The elimination of ROS abrogated the Ang II-induced activation of inflammatory factors, including JNK, TGF-β1, and NF-κB. Ang ІІ binds to a specific receptor, AT1R, which mediates signalling that induces vasocontraction, endothelial dysfunction, and vascular remodeling^36, 37^. Ang ІІ is formed by Ang І cleavage through angiotensin-converting enzyme (ACE) and binds to AT1R to regulate Ang II type 1 receptor associated protein (AGTRAP), while catalytic activation of ACE2 produces Ang (1–7) from Ang I or Ang II^8, 38–40^. Thus, we showed that Ang II bound to AT1R and induced AGTRAP, while it decreased ACE2 expression, leading to inflammation in the atrial cells. Ang II receptor blockers (ARBs) are therapeutic inhibitors of the renin-angiotensin system that exert cardioprotective effects^37, 41^. We assessed the effect of irbesartan, an angiotensin receptor blocker, on the development of inflammation in atrial cells. Irbesartan treatment resulted in the downregulation of AT1R expression and activation in atrial cells, resulting in decreased ROS generation, thereby blocking the JNK/TGF-β1/NF-κB inflammatory signalling pathway.

AT1R is a GPCR that regulates a variety of cellular functions via calcium-dependent pathways^24, 42^. We observed that intracellular calcium concentration increased in Ang ІІ-treated atrial cells. Increased calcium signalling was associated with atrial fibrillation through an inflammation mechanism involving JNK, TGF-β1, and NF-κB. Herein, we demonstrated that calcium signalling regulates inflammation through the Ang ІІ receptor in atrial cells. Elevated calcium concentration was observed upon both acute and chronic exposure to Ang II. Previous studies examined acute and chronic effects of Ang II. Combining the results of previous studies with our calcium experiment results, calcium might be involved in both acute and chronic effects of Ang II. To gain further insights into this mechanism, we examined the possible involvement of the AMPK signalling pathway. AMPK is a crucial protein regulated by calcium signalling. AMPK is well-known as a nutrient and energy sensor that maintains energy homeostasis^43^. Recent studies showed that AMPK is a key regulator of energy metabolism in the heart and during cardiac mitochondrial biogenesis^44, 45^. AMPK, a critical component of metabolic stress response, plays an important role in cardiac ischemia, hypertrophy, and failure through physiological and pathological stress^46–48^. Our data showed that Ang ІІ triggered the activation of AMPK and its downstream effector, ACC, in HL-1 atrial cells. ACC is a negative regulator of fatty acid oxidation^49, 50^. Treatment with the AMPK inhibitor compound C downregulated ACC phosphorylation in atrial cells after Ang II treatment. To investigate the correlation between AMPK and the inflammatory pathway, we also confirmed that JNK activation was inhibited by treatment with compound C in Ang ІІ-stimulated atrial cells. Furthermore, AMPKα2 knockdown also abolished Ang ІІ-activated JNK expression. Subsequently, our data indicated that Ang II-induced TGF-β1 and NF-κB expression was also decreased by blocking AMPK with compound C and AMPKα2 knockdown. In addition, we examined the effect of oxidative stress on the AMPK signalling pathway. AMPK is activated by oxidative stress, which induces hypoxia and increases ROS generation^51, 52^. However, the pathway underlying AMPK activation through ROS during the inflammation process is unknown. NAC treatment inhibited Ang II-induced AMPK stimulation in atrial cells. Finally, inhibition of AT1R by irbesartan treatment resulted in downregulation of the AMPK signalling pathway. Furthermore, CaMKK inhibition also decreased Ang II-induced AMPK stimulation in atrial cells. Collectively, these results showed that blocking ROS generation can prevent the activation of the AMPK signalling pathway during the inflammation process in atrial cells.

In summary, based on the results of this study, we propose a new mechanism of inflammation in atrial cells, which was demonstrated *in vitro* using atrial cells and *in vivo* by assessing the levels of various plasma factors in patients with atrial fibrillation. Considering that silent AF is common in aged population, it is impossible to exclude the probability of silent AF in aged subjects. Therefore, control samples used in this study may have limitation to represent the non-structural heart disease status and without AF rhythm. Several mechanisms are known to be involved in AF, such as focal ectopic firing, re-entry susceptibility, and atrial fibrosis and dilation^53^. Calcium, AMPK, and TGF-β are known to be involved in this process in a complicated manner. Our study reveals two novel findings. First, Ang II-mediated calcium was involved in inflammatory mechanisms via the Ang II-ROS-calcium-AMPK-JNK-TGF-β axis. Second, AMPK acted as a hub molecule, creating a link between Ang II-mediated calcium signalling and JNK-mediated TGF-β induction. The results of this study indicated that Ang ІІ is a key pathophysiological activator of inflammatory mechanisms through binding to its receptor, AT1R. Additionally, we showed that ROS generation by oxidative stress is the main factor underlying the atrial inflammation process. Furthermore, mechanistically, Ang ІІ mediated inflammatory mechanisms involving JNK, TGF-β1, and NF-κB, as well as the AMPK signalling pathway, through ROS activation. Moreover, we demonstrated that AMPK regulates JNK in atrial cells. Our data provide evidence that the AMPK signalling pathway may play an important role in promoting inflammatory mechanisms. It reported that there was no benefit of irbesartan in preventing hospitalization for among AF patients with sinus rhythm. We focused on the effect of irbesartan on atrial inflammation pathway. The meaning of our study should be limited in the effect of irbesartan on inflammatory response and the AMPK signaling, but not in clinical parameters of AF patients, such as reducing cardiovascular events, hospitalization, and first episode of recurrence. Strategies to inhibit these atrial inflammatory mechanisms may represent promising novel therapeutic options for treating inflammation-related diseases, such as atrial fibrillation. In the present study, the concentration of Ang ІІ was slightly increased in patients with atrial fibrillation. We also confirmed that picomolar ranges of Ang II increased TGF-β1 in atrial cells. Although there are differences between *in vitro* and *in vivo* conditions, this result implies that even a tiny increase in AF patients may be physiologically relevant. Further study should be conducted to explain the biological significance of this tiny increase of Ang II.

## Methods

### Reagents

Ang II, N-acetyl-L-cysteine (ROS scavenger), irbesartan (angiotensin receptor blocker), STO-609 (CaMKK inhibitor), compound C (AMPK inhibitor), KN-93 (CaMKII inhibitor), fluo-3 AM, 2′,7′-dichlorofluorescein diacetate (DCFDA), L-ascorbic acid, Claycomb medium, norepinephrine, gelatine, fibronectin, and monoclonal anti-β-actin antibody were purchased from Sigma Chemical Company (St. Louis, MO, USA). Monoclonal antibodies against ACC, CaMKII and CaMKIIδ and polyclonal antibodies against AT1R, TGF-β1, phosphorylated ACC, and phosphorylated CaMKII were purchased from Abcam (Cambridge, MA, USA). Monoclonal antibodies against NF-κB, phosphorylated JNK and AMPKα, and polyclonal antibodies against JNK and AMPKα were obtained from Cell Signaling Technology (Danvers, MA, USA). Foetal bovine serum (FBS), L-glutamine, and penicillin-streptomycin were acquired from Thermo Fisher Scientific (Foster City, CA, USA).

### Cell culture

Mouse HL-1 atrial myocytes were maintained in Claycomb medium supplemented with 2 mM L-glutamine, 100 U/mL penicillin, 100 μg/mL streptomycin, 100 μM norepinephrine in 30 mM L-ascorbic acid, and 10% FBS at 37 °C in a humidified atmosphere of 5% CO_2_. To investigate the effects of Ang ІІ on atrial inflammation, HL-1 cells were seeded in pre-coated 100-mm dishes at a density of 1 × 10^5^ cells/mL with a solution of 0.02% (w/v) gelatine containing 5 μg/mL fibronectin. After 24 h (at >80% confluence), the medium was replaced with FBS-free Claycomb medium. Thereafter, HL-1 cells were treated with Ang ІІ (1 μM) for 24 h, then harvested for further analysis.

### Cell viability assay

Cell viability was assessed using a CellTiter 96 Non-Radioactive Cell Proliferation Assay (MTT) kit (Promega, Madison, WI, USA), based on the reduction of MTT into formazan dye by the action of mitochondrial enzymes. Briefly, HL-1 cells were seeded in 96-well plates at 1 × 10^4^ cells/well and incubated overnight at 37 °C in 5% CO_2_. The cells were treated with indicated concentrations (0, 10, 100, 1000, and 5000 nM) of Ang II for 24 h. The absorbance of each well was measured at 570 nm.

### Cellular metabolic rate

Cell metabolic profile was measured by using a Seahorse XFp Analyzer (Seahorse Biosciences, North Billerica, MA, USA). HL-1 cells were seeded in pre-coated XFp cell culture microplate at 8 × 10^4^ cells/well and incubated overnight at 37 °C in 5% CO_2_. The cells were treated with 1 μM Ang ІІ for 24 h, and the medium was replaced with unbuffered DMEM supplemented with 10 mM glucose, 2 mM L-glutamine, and 1 mM pyruvate (Sigma Chemical). Each cycle included 3 min of mixing, 2 min of incubation, and measurement over 2 min. Three measurements were obtained at baseline and following injection of 1 μM oligomycin, 1 μM FCCP, and 0.5 μM rotenone/antimycin A. Mitochondrial respiration was quantified according to the oxygen consumption rate.

### Assessment of intracellular calcium

Intracellular calcium concentration was measured by detecting the fluorescence of cells treated with a calcium-sensitive indicator fluo-3 AM. Fluorescence was detected using a Zeiss LSM 700 confocal microscope (Zeiss, Oberkochen, Germany). The cells were treated with 5 μM fluo-3 AM in culture medium for 45 min in a CO_2_ incubator. After washing with fresh medium, the cells were incubated without fluo-3 AM for 15 min to ensure complete dye de-esterification. Culture plates were placed on a temperature-controlled microscope stage and were observed under 20× objective. The signal was detected at an excitation and emission wavelength of 488 nm.

### Western blot analysis

The cells were grown in six-well plates. After reaching 60–70% confluence, the cells were serum-starved for 6 h before treatment with selected agents at 37 °C. They were then treated with 1 μM Ang ІІ for 24 h. After treatment, the medium was aspirated. The cells were washed twice with ice-cold phosphate-buffered saline and lysed in RIPA buffer (0.5% deoxycholate, 0.1% sodium dodecyl sulphate, 1% Nonidet P-40, 50 mM NaCl, and 50 mM Tris-HCl [pH 7.4]) containing protease and phosphatase inhibitor cocktails (Sigma Chemical). The supernatants were briefly sonicated, centrifuged for 20 min, and then heated for 10 min at 99 °C. After separation on a 10% sodium dodecyl sulphate-polyacrylamide gel, proteins were transferred onto polyvinylidene difluoride membranes. The membranes were incubated at 4 °C overnight with primary antibodies, after which they were washed six times with Tris-buffered saline containing 1% Tween-20. The membranes were then incubated with horseradish peroxidase-conjugated secondary antibodies for 1 h at room temperature. Anti-β-actin antibody was used to normalize protein loading. The blots were visualized using an ECL solution (Thermo Fisher Scientific). Quantitation was performed by densitometry using Image J.

### Immunoprecipitation

The amount of proteins was determined using the Bradford method. 50 μL of protein A sepharose (Sigma Chemical) was incubated with AMPKα antibody for 2 h at 4 °C. Then, cellular protein (1.5 mg) was added and incubated overnight at 4 °C. After incubation, the samples were washed three times with RIPA buffer (Sigma Chemical). The washed samples were re-suspended in an SDS sample buffer and heated at 100 °C for 15 min prior to electrophoresis.

### Processing of patients with atrial fibrillation

Patients with persistent atrial fibrillation who were admitted for direct current cardioversion (DCCV) to restore sinus rhythm were prospectively screened from December 2015 to July 2016. Plasma samples were serially obtained before DCCV at the time of admission and 1 month after DCCV at the outpatient clinic. As control patients, patients with normal sinus rhythm who underwent electrophysiology study in suspicion for paroxysmal supraventricular tachycardia (PSVT) were prospectively enrolled during the same study period. Eligible patients were enrolled from 2 tertiary medical centres: 11 patients with atrial fibrillation (9 males, average 64 ± 6 years old) and 6 control patients (2 males, age 47 ± 15 years) were enrolled from Seoul National University Hospital, and 15 patients with atrial fibrillation (14 males, age 56 ± 9 years) and 15 control patients (3 males, age 52 ± 16 years) were enrolled from Seoul National University Bundang Hospital. All methods were carried out in accordance with relevant guidelines and all experimental protocols were approved by Seoul National University Bundang Hospital. In addition, informed consent was obtained from all patients.

### Measurement of human plasma levels of Ang ІІ and ROS

Human plasma levels of Ang II were measured by enzyme-linked immunosorbent assay, according to manufacturer’s protocol (Abcam). The plasma concentrations of ROS were quantified using OxiSelect *In Vitro* ROS fluorescence assay kit (Cell Biolabs, San Diego, CA, USA).

### RNA extraction

Total RNA was extracted from 1 × 10^6^ cells/mL using an RNeasy Mini Kit (Qiagen, Valencia, CA, USA) according to the manufacturer’s protocol. RNA concentration and quality were immediately determined using a Nanodrop 2000 (Thermo Fisher Scientific), and aliquots of the total RNA were stored at −80 °C until further use.

### RT-qPCR

Total RNA (50 ng) was used as template to synthesize cDNA using the GoTaq 1-Step RT-qPCR System according to the manufacturer’s instructions (Promega). Reactions were carried out with SYBR green for 40 cycles of denaturation at 95 °C for 10 s, annealing at 60 °C for 30 s, and extension at 72 °C for 30 s using a StepOnePlus Real-Time PCR System (Applied Biosystems, Foster City, CA, USA). The qPCR was performed using the following mouse primers: *Tgf-β1* sense (5′-TTG CTT CAG CTC CAC AGA GA-3′) and antisense (5′-TGG TTG TAG AGG GCA AGG AC-3′), *Tnfα* sense (5′-CCA CCA CGC TCT TCT GTC TA-3′) and antisense (5′-CAC TTG GTG GTT TGC TAC GA-3′), *Il-1β* sense (5′-AAA CGG TTT GTC TTC AAC AAG ATA G-3′) and antisense (5′-ATT CCA TGG TGA AGT CAA TTA TGT C-3′), *Il-6* sense (5′-AGT TGC CTT CTT GGG ACT GA-3′) and antisense (5′-TCC ACG ATT TCC CAG AGA AC-3′), *At1R* sense (5′CTT TAG GAT AAT TAT GGC GAT TGT G-3′) and antisense (5′-GTT AAA ATA CGC TAT GCA GAT GGT T-3′), *Ace2* sense (5′-TGT ATG AAG AGT ATG TGG TCC TGA A-3′) and antisense (5′-CGA AGG TAC GTT CTA CAT CTT CAA T-3′), *Agtrap* sense (5′-AAC TTG AAG GTT ATT CTC CTG GTT C-3′) and antisense (5′-GTA GAT AAT GTC CAG GAA GAT GGT G-3′), and β-actin sense (5′-TGT TAC CAA CTG GGA CGA CA-3′) and antisense (5′-GGG GTG TTG AAG GTC TCA AA-3′). The qPCR on human plasma RNA was performed using the following human primers: *TGF-β1* sense (5′-CCC ACA ACG AAA TCT ATG ACA A-3′) and antisense (5′-ACG TGC TGC TCC ACT TTT AAC T-3′), *TNFα* sense (5′-CTC CTA CCA GAC CAA GGT CAA C-3′) and antisense (5′-AGA CTC GGC AAA GTC GAG ATA G-3′), and β-actin sense (5′-CCA CGA AAC TAC CTT CAA CTC C-3′) and antisense (5′-GGA GCA ATG ATC TTG ATC TTC A-3′). The experiments were performed using three independent biological replicates. Gene expression was normalized to the mRNA expression level of β-actin as an endogenous control, and fold changes in expression were calculated between treatment and untreated control samples.

### *AMPKα2* and *CaMKIIδ* silencing

Cells were seeded in six-well plates, cultured for 24 h to 70% confluence, and then transiently transfected with siRNAs against *AMPKα2* (L-040809, Dharmacon, Lafayette, CO, USA) and *CaMKIIδ* (L-040821, Dharmacon) using Lipofectamine 2000 (Invitrogen, Carlsbad, CA, USA), according to the manufacturer’s protocol. For transfection, 4 µL siRNAs and 8 µL Lipofectamine 2000 were diluted in 500 µL reduced-serum medium and mixed well. The mixture was incubated for 20 min at room temperature and then added dropwise to each culture well containing 1.5 mL serum-free medium (final siRNA concentration, 100 nM). The medium was replaced with fresh complete medium after 6 h of transfection.

### Determination of ROS by confocal microscopy

Intracellular reactive oxygen species (ROS) generation was measured using the fluorescent dye DCFDA, a cell-permeable ROS indicator. Cells were pre-incubated with 10 μM DCFDA for 40 min at 37 °C. Cells were then washed with phosphate-buffered saline. The ROS-mediated fluorescence was observed under a confocal microscope with excitation at 488 nm in real-time.

### Cellular ROS and NADPH oxidase measurements

Intracellular ROS levels were quantified using a ROS-Glo H_2_O_2_ assay kit (Promega). NADPH oxidase activity in HL-1 cell supernatants was analysed by NADP/NADPH quantitation colorimetric kit (BioVision, Milpitas, CA, USA).

### Statistical analysis

A one-way and two-way ANOVA were performed using SPSS version 12.0 (SPSS Inc., Chicago, IL, USA) to determine significant differences among three or more different treatment groups. Data are presented as mean ± standard deviation (SD) of individual experiments. All experiments were performed with at least three independent replicates. The difference between mean values was considered statistically significant at p < 0.05.

## References

[1] Koza Y (2014). Uric acid elevation in atrial fibrillation: is it simply an epiphenomenon or not?. Int. J. Cardiol..

[2] Luong CL (2016). Usefulness of the atrial emptying fraction to predict maintenance of sinus rhythm after direct current cardioversion for atrial fibrillation. Am. J. Cardiol..

[3] Romero-Ortuno R, O’Shea D (2012). Aspirin versus warfarin in atrial fibrillation: decision analysis may help patients’ choice. Age Ageing..

[4] White, C. M. *et al*. Intravenous plus oral amiodarone, atrial septal pacing, or both strategies to prevent post-cardiothoracic surgery atrial fibrillation: the Atrial Fibrillation Suppression Trial II (AFIST II). Circulation. 108 (2003). 10.1161/01.cir.0000087445.59819.6f 12970233

[5] Page RL (2000). beta-blockers for atrial fibrillation: must we consider asymptomatic arrhythmias?. J. Am, Coll. Cardiol..

[6] Tieleman RG (1998). Early recurrences of atrial fibrillation after electrical cardioversion: a result of fibrillation-induced electrical remodeling of the atria?. J. Am. Coll. Cardiol..

[7] Haïssaguerre M (2000). Catheter ablation of chronic atrial fibrillation targeting the reinitiating triggers. J. Cardiovasc. Electrophysiol..

[8] He X (2011). Atrial fibrillation induces myocardial fibrosis through angiotensin II type 1 receptor-specific Arkadia-mediated downregulation of Smad7. Circ. Res..

[9] Ohtsu H (2006). Angiotensin II signal transduction through small GTP-binding proteins: mechanism and significance in vascular smooth muscle cells. Hypertension..

[10] De Mello WC, Danser AH (2000). Angiotensin II and the heart: on the intracrine renin-angiotensin system. Hypertension..

[11] Liu L (2015). Valsartan reduced atrial fibrillation susceptibility by inhibiting atrial parasympathetic remodeling through MAPKs/Neurturin pathway. Cell Physiol. Biochem..

[12] Lu G (2014). Angiotensin II upregulates Kv1.5 expression through ROS-dependent transforming growth factor-beta1 and extracellular signal-regulated kinase 1/2 signalings in neonatal rat atrial myocytes. Biochem. Biophys. Res. Commun..

[13] Oh-hashi K, Kaneyama M, Hirata Y, Kiuchi K (2006). ER calcium discharge stimulates GDNF gene expression through MAPK-dependent and -independent pathways in rat C6 glioblastoma cells. Neurosci. Lett..

[14] Yan J (2013). c-Jun N-terminal kinase activation contributes to reduced connexin43 and development of atrial arrhythmias. Cardiovasc. Res..

[15] Ziolo MT, Mohler PJ (2015). Defining the role of oxidative stress in atrial fibrillation and diabetes. J. Cardiovasc. Electrophysiol..

[16] Lenski M (2015). Arrhythmia causes lipid accumulation and reduced glucose uptake. Basic Res. Cardiol..

[17] Raney MA, Turcotte LP (2008). Evidence for the involvement of CaMKII and AMPK in Ca2 + -dependent signaling pathways regulating FA uptake and oxidation in contracting rodent muscle. J. Appl. Physiol..

[18] Pirkmajer S (2015). Methotrexate promotes glucose uptake and lipid oxidation in skeletal muscle via AMPK activation. Diabetes..

[19] Viollet B (2010). AMPK inhibition in health and disease. Crit. Rev. Biochem. Mol. Biol..

[20] Harada M, Nattel SN, Nattel S (2012). AMP-activated protein kinase: potential role in cardiac electrophysiology and arrhythmias. Circ. Arrhythm. Electrophysiol..

[21] Gu J (2013). Beneficial effects of pioglitazone on atrial structural and electrical remodeling *in vitro* cellular models. J. Mol. Cell. Cardiol..

[22] Abadir PM (2011). Identification and characterization of a functional mitochondrial angiotensin system. Proc. Natl. Acad. Sci. USA.

[23] Emelyanova L (2016). Selective downregulation of mitochondrial electron transport chain activity and increased oxidative stress in human atrial fibrillation. Am. J. Physiol. Heart Circ. Physiol..

[24] Higuchi S (2007). Angiotensin II signal transduction through the AT1 receptor: novel insights into mechanisms and pathophysiology. Clin. Sci. (Lond)..

[25] Madec AM (2013). Losartan, an angiotensin II type 1 receptor blocker, protects human islets from glucotoxicity through the phospholipase C pathway. FASEB J..

[26] Harada M (2015). Atrial fibrillation activates AMP-dependent protein kinase and its regulation of cellular calcium handling: Potential role in metabolic adaptation and prevention of progression. J. Am. Coll. Cardiol..

[27] Park S, Scheffler TL, Rossie SS, Gerrard DE (2013). AMPK activity is regulated by calcium-mediated protein phosphatase 2A activity. Cell Calcium..

[28] Capettini LS (2012). Role of renin-angiotensin system in inflammation, immunity and aging. Curr. Pharm. Des..

[29] Suzuki Y (2003). Inflammation and angiotensin II. Int. J. Biochem. Cell. Biol..

[30] Zhong W (2016). Curcumin alleviates lipopolysaccharide induced sepsis and liver failure by suppression of oxidative stress-related inflammation via PI3K/AKT and NF-κB related signaling. Biomed. Pharmacother..

[31] Choudhury S, Ghosh S, Gupta P, Mukherjee S, Chattopadhyay S (2015). Inflammation-induced ROS generation causes pancreatic cell death through modulation of Nrf2/NF-κB and SAPK/JNK pathway. Free Radic. Res..

[32] Moslehi J, DePinho RA, Sahin E (2012). Telomeres and mitochondria in the aging heart. Circ. Res..

[33] Preston CC (2008). Aging-induced alterations in gene transcripts and functional activity of mitochondrial oxidative phosphorylation complexes in the heart. Mech. Ageing Dev..

[34] Itsumi M (2015). Idh1 protects murine hepatocytes from endotoxin-induced oxidative stress by regulating the intracellular NADP(+)/NADPH ratio. Cell Death Differ..

[35] Ying W (2008). NAD+/NADH and NADP+/NADPH in cellular functions and cell death: regulation and biological consequences. Antioxid. Redox. Signal..

[36] Ram CV (2008). Angiotensin receptor blockers: current status and future prospects. Am. J. Med..

[37] Zografos T, Katritsis DG (2010). Inhibition of the renin-angiotensin system for prevention of atrial fibrillation. Pacing Clin. Electrophysiol..

[38] Huang H, Zhou J, Cui Z, Wang B, Hu Y (2015). Angiotensin II type 1 receptor-associated protein plays a role in regulating the local renin-angiotensin system in HSC-T6 cells. Mol. Med. Rep..

[39] Horiuchi M, Iwanami J, Mogi M (2012). Regulation of angiotensin II receptors beyond the classical pathway. Clin. Sci. (Lond)..

[40] Tan AY, Zimetbaum P (2011). Atrial fibrillation and atrial fibrosis. J. Cardiovasc. Pharmacol..

[41] Mahmood A (2015). Differential effects of β-blockers, angiotensin II receptor blockers, and a novel AT2R agonist NP-6A4 on stress response of nutrient-starved cardiovascular cells. PLoS One..

[42] Liu K (2010). A multiplex calcium assay for identification of GPCR agonists and antagonists. Assay Drug Dev. Technol..

[43] Hardie DG, Ross FA, Hawley SA (2012). AMPK: a nutrient and energy sensor that maintains energy homeostasis. Nat. Rev. Mol. Cell Biol..

[44] Dyck JR, Lopaschuk GD (2006). AMPK alterations in cardiac physiology and pathology: enemy or ally?. J. Physiol..

[45] Niemann B (2013). Age and obesity-associated changes in the expression and activation of components of the AMPK signaling pathway in human right atrial tissue. Exp. Gerontol..

[46] Voelkl J (2016). AMP-activated protein kinase α1-sensitive activation of AP-1 in cardiomyocytes. J. Mol. Cell. Cardiol..

[47] Zaha VG, Young LH (2012). AMP-activated protein kinase regulation and biological actions in the heart. Circ. Res..

[48] Kim GE (2015). LKB1 deletion causes early changes in atrial channel expression and electrophysiology prior to atrial fibrillation. Cardiovasc. Res..

[49] Kim N (2016). AMPK, a metabolic sensor, is involved in isoeugenol-induced glucose uptake in muscle cells. J. Endocrinol..

[50] Arad M, Seidman CE, Seidman JG (2007). AMP-activated protein kinase in the heart: role during health and disease. Circ. Res..

[51] Mungai PT (2011). Hypoxia triggers AMPK activation through reactive oxygen species-mediated activation of calcium release-activated calcium channels. Mol. Cell Biol..

[52] Sook SH (2014). Reactive oxygen species-mediated activation of AMP-activated protein kinase and c-Jun N-terminal kinase plays a critical role in beta-sitosterol-induced apoptosis in multiple myeloma U266 cells. Phytother. Res..

[53] Nattel S, Dobrev D (2016). Electrophysiological and molecular mechanisms of paroxysmal atrial fibrillation. Nat. Rev. Cardiol..

